# Updates on feline aelurostrongylosis and research priorities for the next decade

**DOI:** 10.1186/s13071-016-1671-6

**Published:** 2016-07-07

**Authors:** Hany M. Elsheikha, Manuela Schnyder, Donato Traversa, Angela Di Cesare, Ian Wright, David W. Lacher

**Affiliations:** School of Veterinary Medicine and Science, University of Nottingham, Sutton Bonington Campus, Leicestershire, LE12 5RD UK; Institute of Parasitology, Vetsuisse Faculty, University of Zurich, Winterthurerstrasse 266a, Zürich, 8057 Switzerland; Faculty of Veterinary Medicine, University of Teramo, Teramo, Italy; Withy Grove Veterinary Surgery, 39 Station Rd, Bamber Bridge, Preston, PR5 6QR UK; Division of Molecular Biology, Center for Food Safety and Applied Nutrition, United States Food and Drug Administration, Laurel, MD USA

**Keywords:** *Aelurostrongylus abstrusus*, Aelurostrongylosis, Cat, Lungworm

## Abstract

Feline aelurostrongylosis, caused by the metastrongyloid nematode *Aelurostrongylus abstrusus*, is an important gastropod-borne parasitic lung disease in cats. Infection with *A. abstrusus* is widespread globally, but the increasing awareness of this parasite and the advent of more sensitive diagnostics have contributed to the apparent increase in its prevalence and geographic expansion. Clinical features may range in severity from subclinical to life-threatening respiratory disease. Parasitological standard techniques, such as visualization of the nematode first larval stage in faecal and respiratory (bronchial mucus or pleural fluid) samples, remain the mainstays of diagnosis. However, diagnosis is evolving with recent advances in serological and molecular testing, which can improve the time to initiation of effective anthelmintic therapy. Despite numerous anthelmintics that are now available as treatment options, the role of host immunity and lifestyle factors in selecting cats that may benefit from more targeted anthelmintic prophylaxis or treatment practice remains unclear and is likely to guide therapeutic choices as newer data become available. This review summarizes the biology, epidemiology, pathophysiology, diagnosis and treatment options currently available for feline aelurostrongylosis.

## Background

Feline aelurostrongylosis is an important respiratory disease affecting domestic cats worldwide [1–3]. It is caused by the metastrongyloid nematode *Aelurostrongylus abstrusus* Railliet, 1898 (Strongylida: Angiostrongylidae), the “cat lungworm”, which resides in the bronchioles and alveolar ducts of the feline definitive host, i.e. the domestic cat *Felis silvestris catus*. Also, there have been several, though still equivocal, reports on *A. abstrusus* in other species of felids (see host-specificity section). The nematode can elicit various clinical manifestations, ranging from minimal respiratory signs to interstitial bronchopneumonia, dyspnoea and respiratory distress in heavy infections. Even though *A. abstrusus* is considered by many practitioners sporadic and relatively non-pathogenic, the last few years have witnessed increasing awareness of its impact on feline health [3–6]. Depending on the life style (indoors, outdoors), geographic origin and methods used for diagnosis, recorded prevalence in cats varies widely from 1.2 % in owned cats [7] to 50 % in free roaming cats [8].

Other lungworm species, such as *Oslerus rostratus* have been recorded in domestic cats [3]. For instance, a mixed infection of *A. abstrusus* and *O. rostratus* has been reported in a domestic cat from Spain [9]. The presence of adults and first-stage larvae (L1s) of *O. rostratus* in a domestic cat indicates a role for this felid host as definitive host. However, because *O. rostratus* is a parasite of wild felids, there is a speculation that the domestic cat is an accidental host. Other metastrongyloids, such as *Troglostrongylus brevior* and, to a lesser extent *Troglostrongylus subcrenatus* (recorded only in a single cat), have also been recently reported in domestic cats [3, 10, 11]. However, this review focuses only on *A. abstrusus*.

The cat lungworm has an indirect life-cycle that requires invertebrate gastropods as an intermediate host within which the first-stage larvae (L1s) mature to the infective third-stage larvae (L3s) [12]. Cats can become infected by ingesting intermediate or paratenic hosts [13]. After ingestion, the larvae migrate to the lungs via the lymphatic vessels and mature into adult stages [13]. Detection of adult *A. abstrusus* can be challenging because of their embedment in the lung parenchyma; different methods and attempts to correlate adult worm burdens with faecal larval count have previously been used with varying success [8, 14, 15]. The Baermann technique is the routinely used diagnostic method for identification of L1 in the faeces [2], but not without limitations. There is still a need to develop better methods that allow sensitive and specific detection of the infection and the timely initiation of appropriate anthelmintic therapy.

In this article, an account of recent advances in knowledge of biology, epidemiology, manifestations of disease, diagnostics and treatment options currently available for feline aelurostrongylosis is provided.

### Life-cycle and transmission

**Fig. 1:**
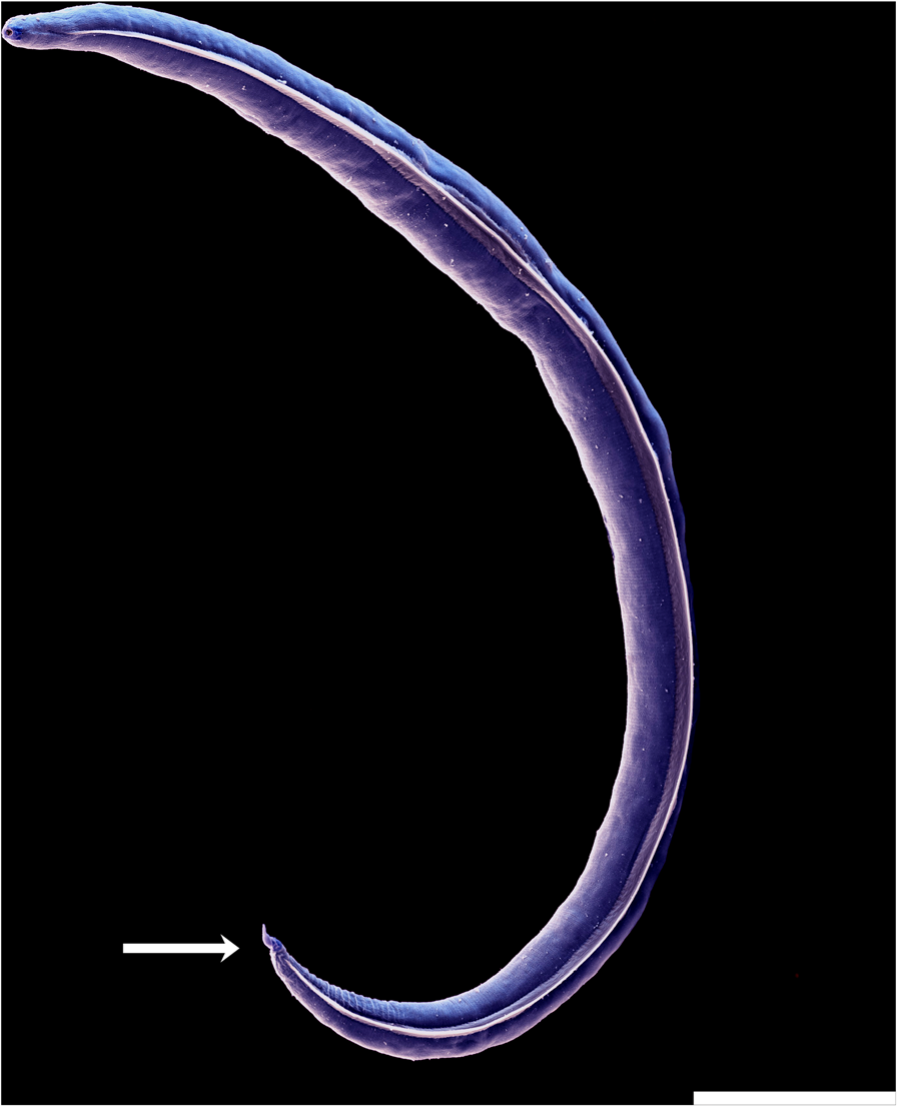
Scanning electron micrograph of *Aelurostrongylus abstrusus* first-stage larva (L1) isolated from cat faeces by Baermann technique. Larva measures approximately 360 to 400 μm in length and the tail ends in a unique sinus wave-shaped kink with a dorsal subterminal spine (*arrow*). Image courtesy of Bayer Animal Health. *Scale-bar*: 50 μm

*Aelurostrongylus abstrusus* has an indirect life-cycle with cats as definitive hosts and snails or slugs as intermediate hosts. Adult worms reside in the alveolar ducts and terminal respiratory bronchioles of the felid host. Following fertilization the oviparous females lay eggs that hatch within the pulmonary ducts and alveoli. The L1s (Fig.1) migrate via the bronchial/tracheal escalator to the pharynx, are swallowed and passed in the cat faeces to environment. L1s penetrate snails or slugs, where they develop to L3s. Mice, birds, reptiles and amphibians may serve as paratenic hosts by ingestion of infected gastropods [12, 16]. The fact that the Mediterranean edible snail *Helix aspersa* can shed infective L3s of *A. abstrusus* in the environment [17], and the demonstration of snail-to-snail transmission of *A. abstrusus* L3s from experimentally infected to naïve *H. aspersa* hosts [18] provided new insights, though still under laboratory conditions, into potential alternative pathways for the transmission of *A. abstrusus*. Cats become infected by either eating snails or paratenic hosts; once ingested, the infective L3s [19] penetrate the intestinal mucosa of the definitive hosts and via lymphatics reach to the lungs where they develop into sexually mature adults. The prepatent period lasts approximately 35–48 days [14, 20]; the excretion of L1s in faeces may fluctuate, being the highest around 10–14 weeks after infection and lasting in individual cats for several months up to more than a year [20, 21].

### Host-specificity

*Aelurostrongylus abstrusus* is the so-called “cat lungworm” because the domestic cat is considered its natural host [1]. There are however some reports of infection by *A. abstrusus* in other species of felids. Recent studies have demonstrated that *A. abstrusus* may infect the European wildcat (*Felis silvestris silvestris*) in certain geographical areas. In particular, *A. abstrusus* has been unequivocally identified in European wildcats examined in the central and southern regions of Italy even with high prevalence rates (62.5 %) and in association with severe lung damage [22].

One might argue that there is no definite evidence that *A. abstrusus* infects wild felids, but rather records reflect some misidentifications with other parasites and/or a lack of comprehensive description of nematodes found in wild felids [23]. For instance, L1s found in the faeces of lions (*Panthera leo*) were identified as “*Aelurostrongylus* sp.”, despite they had a length (~250–300 μm) and a width (12.5–15 μm) more likely consistent with *Troglostrongylus* spp. [24]. L1s collected from one Amur cat (*Felis bengalensis euptilurus*) were identified as *A. abstrusus*, but they had a length (~340–360 μm) and a width (~15 μm) again consistent with *Troglostrongylus* spp. [25]. No information is reported on the morphological and morphometric features used to identify the L1s from a cheetah (*Acinonyx jubatus*) and the corresponding adult parasites obtained after an experimental infection, and no descriptions of the parasitic stages found in that study are provided [26]. But these findings might indicate the cheetah’s ability to carry patent *A. abstrusus* infection. Analogously, no description is present in the reference [27] citing a checklist from Brazil [28], which reports *A. abstrusus* in a jaguarundi (*Herpailurus yagouarondi*). Although the length (~370–395 μm) is consistent with *A. abstrusus*, the L1s identified as *A. abstrusus* from the Eurasian lynx (*Lynx lynx*) [29] are wider (up to 25 μm) than the values considered diagnostic for *A. abstrusus* [3, 30]. Finally, although the brief description of histological findings is consistent with *Aelurostrongylus* spp., there is no description of parasites identified as *A. abstrusus* found at the necropsy of one European wildcat (*Felis silvestris silvestris*) from Portugal [31]. What derives variations in the size of L1s among these different definitive host species remains unknown, but variation in body size of nematodes may just reflect adaptation of the parasite to different physiological environments (i.e. the amount and nature of available nutrients) and host immune defences of these different felid hosts, in analogy to what has been described in oxyurid nematodes [32, 33]. However, it cannot be excluded that the larvae found in the aforementioned species of wild felids were other poorly known species of the genus *Aelurostrongylus*, e.g. *A. falciformis* and *A. pridhami*, which usually infect mustelids [34], or eventually *Aelurostrongylus* spp. yet to be described.

### Epidemiology and the impact of lifestyle and climate

The nematode may be harbored by cats regardless of their habitat, lifestyle, breed and sex but privately owned animals, cats living indoor or with few chances to access outdoor, are less prone to be infected by *A. abstrusus*. In contrast, animals living outdoors, with a remote lifestyle and allowed to hunt, have enhanced opportunities to ingest molluscs and/or prey [3, 35, 36]. Surveys carried out in Brazil [37] and Italy [4] have indicated that free-ranging animals and young cats may be significantly more often infected with *A. abstrusus*. Conversely, data from surveys in Australia [38] and eastern Europe [5, 8, 39] have shown that *A. abstrusus* is more prevalent in adult cats that are likely to have a greater hunting ability and lifespan, possibility cumulative and higher chances of ingesting L3s. A very recent large-scale survey, carried out in northern and central Italy and involving more than 800 cats, has confirmed that both young and adult animals were infected by *A. abstrusus* and that cats younger than 1 year were more at a risk of infection with *T. brevior* rather than with *A. abstrusus* [40]. Another study from Sardinia, Italy, reported that age and sex do not seem to be risk factors for *A. abstrusus* infection [35].

A multicenter study conducted in nine veterinary faculties across Europe revealed outdoor access and geographic locality as risk factors for *A. abstrusus* infection in cats [41]. Also, some areas of southern Europe may offer suitable ecological and epidemiological conditions for the occurrence of felid respiratory parasites [36]. It has been suggested that the dispersion of mollusc-transmitted parasitoses is triggered by climate changes [42, 43]. At the moment no specific data are available for *A. abstrusus*, but with similar biological cycles, the same factors involved in the apparent expansion of other mollusc-borne parasites (e.g. *Angiostrongylus* spp.) would likely also have an effect on *A. abstrusus* [43, 44]. As environmental factors, i.e. temperature, moisture and water availability, may influence the development and survival of gastropods and of nematode larvae in their mollusc intermediate hosts [42, 43], this could also be true for *A. abstrusus*. Accordingly, the higher the average temperature the higher the rate of larval development of *A. abstrusus* in *H. aspersa* snail, i.e. a common and efficient intermediate host of the cat lungworm [45]. Thus, increasing temperatures might truly contribute to the apparent spread of this nematode (and other metastrongyloids) in Europe [46]. Moreover, a key role in the apparent expansion of *A. abstrusus* could be played by *H. aspersa* itself*.* This mollusc is one of the most widely spread land snails in the world [47] and has been deliberately or accidentally imported in several regions (e.g. by the movement of plants and vegetables) where it is now considered a pest outside its native Mediterranean range [48]. This snail is also extensively farmed for human consumption in several countries [47], usually in outdoor pens, which may increase the risk for the biological interactions between snails, lungworms and suitable vertebrate hosts. A key example on how the epidemiology of *A. abstrusus* is likely changing is given by the recent study carried out in Italy from 2014 to 2015 on the occurrence of larvae of cats examined with microscopic and genetic methods [40]. This study has shown that *A. abstrusus* is the most common lung parasite in both mono- and poly- specific infections in domestic cats, with a prevalence rate of up to 17 % in different geographical regions, while until a decade ago, *A. abstrusus* was considered sporadic in Italy (and in Europe as well), and most records were either single clinical cases or accidental descriptions of larvae in the faeces of cats [2]. Nonetheless, these single cases and the development of better diagnostic tools have contributed to increased scientific interest and therefore increased disease awareness, allowing the growth of our knowledge of this parasite. Improving the understanding of ecological factors that drive the growth and survival of *A. abstrusus* in the environment could assist in predicting and preventing exposure of cats to this parasite.

### The prevalence

*Aelurostrongylus abstrusus* has a cosmopolitan distribution and has been recorded in nearly all countries in Europe, frequently in Australia and the Americas, and sometimes in Asia and Africa [1]. For instance, prevalence rates of 14 to 39.2 % have been described in regions of Australia, e.g. Tasmanian Midlands/King Island and Christmas Island, respectively (reviewed in [6]). In the USA, prevalence rates of 6.2 % in New York and of 18.5 % in Alabama have been reported in shelter and stray cats, respectively, while in Argentina the parasite has been recorded in 2.6 % of examined stray cats (reviewed in [6]). The presence of *A. abstrusus* has been shown in cats from different European countries with relatively higher prevalences, e.g. 39.7–50 % in Albania, 1.8–22.4 % in Italy, 0.38–22 % in Croatia, 0.5–15.3 % in Germany, 3.6–10.6 % in Great Britain, 2.6 % in Holland, 14.5 % in Hungary, 17.4 % in Portugal, 5.6 % in Romania, 1 % in Spain, and in clinical cases in Belgium, France, Ireland, Norway, Poland and Turkey (reviewed in [46]). In Greece, 125 stray cats were examined in four geographical locations in continental and insular Greece, and a prevalence of 17.4 % in Athens, 2.9 % in Crete, 7 % in Mykonos and 8 % in Skopelos islands has been recorded using both Baermann and molecular methods [49]. In Denmark, the parasite has been detected in outdoor cats from different region of the country with a prevalence range of 13.6–15.6 % by performing a perfusion and lung digestion technique of dissected feral and domestic cats [15]. Prevalence data may vary depending upon the clinical materials and diagnostic method used, i.e. the Baermann technique and molecular methods (performed on faeces, bronchoalveolar lavage (BAL) or lung material) or the lung digestion in dissected cats, and upon the analysed cat population, with free-roaming stray cats presenting the highest prevalence irrespective of the country. This variance renders the comparison among prevalence data challenging.

### Molecular phylogenetics

Each cluster of ribosomal DNA (rDNA) contains external transcribed spacer (ETC), 18S rDNA, internal transcribed spacer 1 (ITS1), 5.8S rDNA, internal transcribed spacer 2 (ITS2) and 28S rDNA. These genes and spacer regions have been used as molecular markers for the genetic make-up of *A. abstrusus*. Among them, the 18S rRNA and 28S rRNA [50] and ITS2 gene sequences were used for the genetic characterization of *A. abstrusus* [51]. Based on the variable ITS2 region, a nested PCR test with specificity of 100 %, was established for diagnosis of this parasite from faeces and pharyngeal swabs [52]. Molecular approaches enabled the detection of L3 of *A. abstrusus* in striped field mice (*Apodemus agrarius*), suggesting the role of this naturally infected paratenic host in the biology, ecology and epidemiology of *A. abstrusus* [53]. The recent sequencing of the mitochondrial genome of this parasite [54] may increase our understanding of the unique pathogenic properties of *A. abstrusus* and speed up the development of more molecular diagnostic tools.

**Fig. 2:**
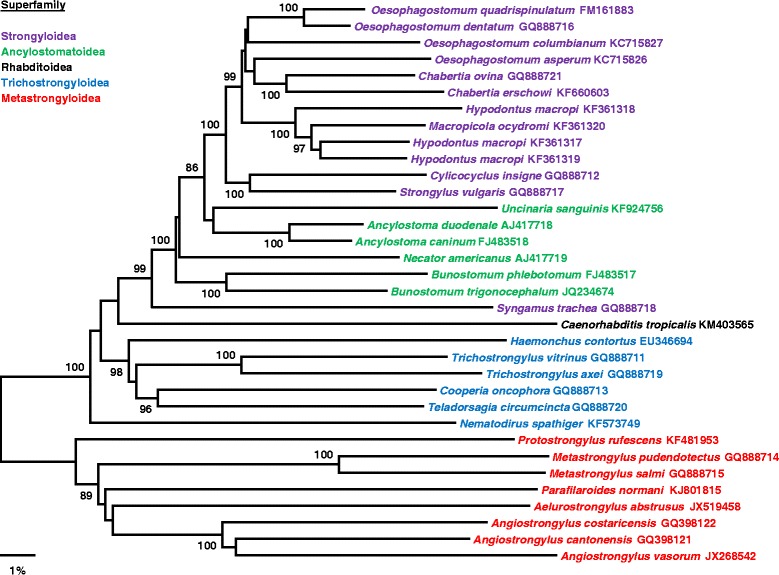
Phylogenetic relationships among 32 species of nematodes of the order Rhabditida. This neighbor joining tree was constructed using the concatenated translated amino acid sequences of the 12 mitochondrial protein-encoding genes and a p-distance matrix. Bootstrap values greater than 85 % are given at the internal nodes. Taxa are color-coded based on their superfamily designation (see key for details) and are labelled with their species and GenBank accession number. The alignments were done using the MegAlign Pro module of the Lasergene software package (DNASTAR, Inc) and the phylogenetic tree was generated using MEGA. A decrease in branch support of this group in the combined analyses involving the mitochondrial genomes is probably related to intrinsic features of these genomes, which may hamper the establishment of homologies during the alignment of relatively large matrices

Phylogenetic analysis of *A. abstrusus* based on ITS2 sequences showed that *A. abstrusus* clustered with other lungworm species of veterinary importance (e.g. *Metastrongylus* spp., *Elaphostrongylus* spp.) [51]. A similar finding was obtained based on the concatenated amino acid sequence data for all protein-encoding mitochondrial genes [54]. To gain further insight into the precise phylogenetic position of *A. abstrusus* we constructed more expanded phylogenetic trees, which were based on the mitochondrial genome sequences of *A. abstrusus* and related species. The analyses of combined mitochondrial genome sequences increased the resolution of phylogenetic analyses and allowed us to confidently define a phylogenetic position for *A. abstrusus* within the Metastrongyloidea (Fig. 2). *Aelurostrongylus abstrusus* clustered with and formed a monophyletic group with *Angiostrongylus costaricensis* and *A. vasorum*. Comparing whole mitogenome sequences has the potential to further increase the resolution of the phylogenetic analysis, particularly where recent divergence, slow genome evolution or rapid speciation has resulted in limited sequence variation. Also, as indicated in Table 1, the cytochrome *c* oxidase subunit I (*cox*1) gene involved in energy metabolism was the most conserved gene, while *nad*2 and *nad*6 were the most polymorphic among the species analysed. If the pattern of observed amino acid sequence variation holds for within species, then one would expect low levels of diversity for *cox*1 among different *A. abstrusus* strains, while *nad*2 and *nad*6 should be considerably more variable. Phylogenetic studies designed to track *A. abstrusus* genotypes over different environmental regions may shed some light on the relevance and extent of environmental expansion and transfer of this parasite to new localities or new hosts.

**Table 1:** Variation among nematode species listed in the phylogenetic tree observed at mitochondrial loci

Locus	Average nucleotide similarity (%)	Average amino acid similarity (%)
*atp*6	79	81
*cox*1	85	93
*cox*2	82	87
*cox*3	82	87
*cytb*	79	81
*nad*1	78	77
*nad*2	71	62
*nad*3	81	80
*nad*4	76	75
*nad*4L	79	76
*nad*5	74	71
*nad*6	71	63
*rrn*L	80	na
*rrn*S	83	na
Overall	79	78

*Abbreviation*: *na* not applicable

### Clinical features of *A. abstrusus* infection

Clinical manifestations of feline aelurostrongylosis range widely from subclinical [1] to a variety of respiratory signs, such as respiratory distress including dyspnoea, open-mouthed abdominal breathing, coughing, wheezing, sneezing and mucopurulent nasal discharge [4, 35, 55]. Pneumothorax and pyothorax secondary to *A. abstrusus* infection has been reported in a 14-week-old kitten exhibiting vomiting, diarrhoea and pyrexia, and it was speculated that *Salmonella typhimurium* was carried by L3s from the intestine to the lungs in a “Trojan horse” mechanism [56]. Such non-specific clinical patterns require a high level of clinical awareness of the disease in order to guide the prompt institution of treatment.

### Why do infected cats die?

Death can occur in severe cases especially in young, debilitated or immunosuppressed animals [2, 43, 57]. *Aelurostrongylus abstrusus* has been implicated in causing what is called a hyperinfection syndrome, such as in the case of a 2-month-old feral kitten, from the UK, which died due to verminous pneumonia and enteritis. Before death, the kitten exhibited both respiratory and intestinal manifestations and on post mortem examination *A. abstrusus* eggs and larvae were present in alveoli, along with adult worms in small bronchioles. Small intestinal mucosa also contained a large number of larvae, which was speculated to be sufficient to cause diarrhoea [58]. Furthermore, a report from the USA described infection of two cats with granulomatous interstitial pneumonia due to *A. abstrusus* and contemporaneous infection of the intestinal tract with *A. abstrusus*, with small numbers of larvae observed on histological examination in the colonic crypts and, occasionally, on the surface of the colon [59]. Larval burden in the colon in both cats was not considered to be sufficient to induce enteric disease, which explains why the cats did not exhibit diarrhoea. The cause of death of one cat was attributed to hypertrophic cardiomyopathy secondary to hyperthyroidism and a mild, subclinical, verminous pneumonia. The second cat died due to cor pulmonale secondary to severe verminous pneumonia due to *A. abstrusus* [59].

### Pathogenesis of feline aelurostrongylosis

#### Inflammatory pathology

Significant advances have been made in deciphering the pathogenesis of *A. abstrusus* infection. The pathological damage caused by *A. abstrusus* is attributed to the host inflammatory reaction in response to the presence of different stages of *A. abstrusus* in the respiratory tract. Adult stages can be found deeply embedded within and hard to be teased out of the lung parenchyma [13, 14, 60]. However, inflammatory reactions surrounding adult stages are rarely found [14, 58, 61]. In contrast, numerous migrating immature stages and the offspring of adult worms, larvae and eggs, are regularly surrounded by granulomata and inflammatory cells, resulting in prominent pathological changes [1, 14, 62] and reduction in the available surface area for gas exchange [61].

#### Immune response

The tissue damage seen by histological examination may be interpreted as morphological evidence of the parasite’s ability to subvert immune responses. The involvement of the immunological host defence is indicated by hyperplasia of peribronchial lymph nodes [63, 64] and enlargement of lymph nodes [14, 65]. Individual variations of pathological changes observed in naturally and experimentally infected cats could be due to varying numbers of ingested L3s, with more obvious reactions to increasing numbers of L3s [14, 55, 64], but also to the heterogeneity of individual immunological responses [14]. Importantly, cats inoculated at regular intervals with small doses of infectious L3s can be protected against a challenging large dose of infective larvae [66].

#### Vascular pathologies

In addition to the damages they cause to the lung tissue, eggs, larvae and inflammatory exudate have been suggested to cause bronchiolar muscular hypertrophy, and hypertrophy and hyperplasia of the smooth muscle of the pulmonary arteries, gradually obstructing the bronchiolar system [67, 68] and inducing increased peripheral vascular resistance [69]. Thickening of the pulmonary vessels’ media has been observed in cats 4–18 weeks after infection along with massive inflammatory reactions that correlated with the severity of the arterial lesions [70]. These changes were diminished after 2 years, but arteriopathy remained. Arterial change was also suggested to be due to the effect of excretory or secretory products of *A. abstrusus* on the vessels ([71] cited in [67]) or to the resistance of the blood flow through lung parenchyma as a consequence of increased pulmonary pressure [67]. This latter alteration, based on comparable vascular changes with *Dirofilaria immitis* infection in cats, was suggested to cause pulmonary hypertension [61]. Vasoconstriction induced by mast cells and histamine release, promoting pulmonary vascular resistance was also hypothesised [72]. The fact that *A. abstrusus* infection was the most frequent finding in cats dying during anaesthesia supports the negative influence of pulmonary hypertension (in analogy with people having pulmonary hypertension). Sedation or anaesthesia may reduce cat’s ability to compensate for diminished gas exchange surface area, compromising lung perfusion and ventilation, which can lead to hypoxia, systemic hypotension and cardiovascular arrest [61, 72].

### Diagnostic tools for detection of *A. abstrusus*

#### Direct parasitological findings

Copromicroscopic examination is still the mainstay of the diagnosis of *A. abstrusus* infection and is achieved via the detection of typical L1s in the faeces of infected cats. Direct faecal smears and classical sedimentation and flotation methods are less-sensitive and are impaired by the solution used and length of time needed to process the sample, as high specific gravity concentrated solutions can cause osmotic larval damage. Larvae become dehydrated and/or sink and they may lose morphological details, and, as a consequence, become hard to detect and differentiate [43]. The most frequently used method to diagnose cat aelurostrongylosis is the isolation of L1s from faeces through Baermann technique [73], but this requires 12–24 h and fresh faeces and specific skill in discriminating L1s [38, 43]. Furthermore, the Baermann method cannot detect infections in the pre-patent period and when larvae are not shed. Shedding may be intermittent and/or absent, even in presence of clinical signs, especially in chronically infected cats and cats with reinfections, which show sporadic shedding patterns [1, 14, 20, 67]. FLOTAC is more sensitive and less time-consuming than Baermann, McMaster and Wisconsin techniques, and does not rely on larval migration, an essential condition for Baermann technique; hence it has the added value of allowing identification of L1s in old preserved or frozen faeces [74].

First-stage larvae of *A. abstrusus* should be identified based on their length and on the morphological attributes of the anterior and posterior ends. Most descriptions in the scientific literature report that L1s of *A. abstrusus* are ~360–400 μm long, although shorter larvae, down to ~ 300 μm, have been described in cases of aelurostrongylosis confirmed upon histological and genetic analyses [3]. Therefore, the identification of *A. abstrusus* L1s should be based also on head and tail morphological features. These larvae have a rounded head with terminal oral opening, and a kinked (S-shaped) tail with distinct knob-like or small finger-like projections at the tip with cuticular spines, a deep dorsal incisure and a ventral incisure [3, 11, 36]. L1s of *A. abstrusus* need to be discriminated from those of other lungworms (e.g. *Troglostrongylus* spp., *O. rostratus*), from ancylostomatid hookworm larvae that may be present in samples that have been incubated in order to allow eggs to embryonate, and from free-living nematodes which can be present in samples collected from the soil [11, 36, 43].

While key features allowing this discrimination have recently been reported [3, 11, 43], there is a paucity of information on the morphological and morphometric differences between L1s of *A. abstrusus* and *Angiostrongylus chabaudi*. This latter metastrongyloid was described last century in six European wildcats from central Italy and remained unknown until the past 2 years, when it has been described in a very few animals from Italy (reviewed in [36]). However, at the moment there is no evidence that this parasite may mature and reproduce in the domestic cat, as no any record of patent angiostrongylosis by *A. chabaudi* is available in the literature [36]. Nonetheless, a case of patent infection by *A. chabaudi* has been recently described from one European wildcat in Greece [75]. This article reported the only available description of L1s of *A. chabaudi*, which displayed a typical *Angiostrongylus*-like morphology, including body size (length 362–400 μm, width 15–18.5 μm), and a kinked tail with a dorsal spine and a notch [75]. Further data are necessary to provide the conclusive features that allow unequivocal differentiation between larval *A. abstrusus* and *A. chabaudi* in cat faeces, pending the demonstration that *F. s. catus* may also act as definitive host of *A. chabaudi*.

Respiratory samples, e.g. tracheal swabs or wash, BAL, pleural effusions and expectorated material, may be microscopically examined for the presence of *A. abstrusus* L1s. Recently, cytological evaluation of fine needle aspirate of sonographically affected lung has been reported in a domestic shorthair cat from Alabama, USA [76]. However, these methods have inherent limitations in terms of risks for the animal’s health while obtaining the material, requirement of general anesthesia combined with low sensitivity in the absence of significant pulmonary tissue involvement [43, 77]. Due to the intermittent faecal excretion of L1s, the simultaneous use of Baermann and BAL testing has been suggested [38]. Fine needle aspiration of the lungs has been performed in two cats exhibiting severe dyspnea and the cytological examination of the aspirate revealed *A. abstrusus* larvae [78].

#### Laboratory findings

Blood analyses are among the first diagnostic measures performed for diagnostic work-ups in sick cats presented to the clinician. It is therefore of importance to recognize parameters which may be altered in cats infected with *A. abstrusus*, although not pathognomonic. Laboratory findings such as leucocytosis [57], eosinophilia [57, 67, 79], anaemia [79] and hypoalbuminaemia [80] have been described in case reports. Eosinophilia seems to be the most persistent finding, presumably due to the constant antigen stimulation caused by the presence of the parasites [14]. Endoparasites are also known to induce lymphocytic immune reactions with IgE production and lymphocytosis [81]. In addition, blood gas analysis performed on clinically affected cats identified respiratory acidosis (blood pH < 7.34 and pCO_2_ > 36) in three out of four cats infected with *A. abstrusus* and has been suggested to aid a better management of heavily affected cats with respiratory acidosis [79].

The temporal changes in these parameters have been identified in experimentally infected cats. In general, eosinophilia (also in bone marrow) and leucocytosis were found to be the most frequent changes between 2 and 4 weeks post-inoculation [1, 14], and remained largely out of reference ranges during the course of infection. Interestingly, leucopenia was also observed between 6 and 10 weeks post-infection [1]. Mild anaemia has been detected quite often; while basophilia, monocytosis and lymphocytosis were only occasionally present. Chemistry values were always within reference ranges [14]. Although not routinely performed, the increased prothrombin time, reduced activated partial thromboplastin and thrombin time, and reduced amount of fibrinogen, have been found to occur in an irregular manner during the first 6 weeks after infection [14]. Furthermore, serum electrophoresis identified mild changes, such as reduction of α globulins (20, 34, 48, and 133 days post infection) and an increase of β1 globulins (20 and 34 days post-infection) [82].

#### Molecular diagnostics

In the past few years molecular assays have been developed for a DNA amplification-based diagnosis of cat aelurostrongylosis. The first technique developed was a nested PCR based on genetic markers within the rDNA of *A. abstrusus*. This assay showed a 100 % specificity and a sensitivity up to ~97 % on a panel of faecal (i.e. faeces, floatation supernatant, Baermann sediment) and pharyngeal swab samples from infected cats [52]. Importantly, this assay was able to unveil cats that scored negative upon the classical diagnostic methods and has been powerful in field studies [52, 83]. Later, a duplex PCR based on ITS2 marker within rDNA was developed to discriminate faecal L1s of *A. abstrusus* and *T. brevior* in a single cat with a mixed infection [84]. Very recently, a triplex semi-nested PCR assay has been validated for the simultaneous discrimination of *A. abstrusus*, *T. brevior* and *Angiostrongylus chabaudi*. This method proved to be highly promising for basic and applied studies on these nematodes [85]. In general, genetic assays proved to be highly efficient when applied on pharyngeal swabs that represent the most suitable sample for the molecular diagnosis of *A. abstrusus* in terms of sensitivity and for reasons of practicality or convenience. In fact, the use of swabs overcomes difficulties of adequate faecal collection in the field, and also overcomes laborious DNA extraction from faeces and the presence of PCR-inhibitors in faecal samples.

Appropriate collection of faecal samples from cats for routine analyses, i.e. collection of fresh faecal material over 3 days, as recommended to increase sensitivity of the diagnostic methods, may be arduous, since free-roaming animals typically defecate outdoors. Taking this into count and considering the subtle nature of chronic lungworm infection and the lack of specific clinical signs, serological methods for the detection of *A. abstrusus* would be of great help. However, there are no commercially available serological tests for diagnosis of aelurostrongylosis. Older serological assays (e.g. indirect fluorescent antibody test, IFAT) have been limited by cross-reactivity with antigens of other endoparasites and poor discriminatory properties between past and present infections [86]. Recently, an IFAT able to detect antibodies against *A. abstrusus* in sera from both experimentally and naturally infected cats showed to be promising in terms of sensitivity and specificity [87]. First results obtained with an enzyme-linked immunosorbent assay (ELISA) for the detection of specific antibodies have been described [88]; such a test would be highly suitable for mass-screening and seroepidemiological studies, and may also be adopted and further developed for serological diagnosis of clinical cases.

#### Radiological manifestations

**Fig. 3:**
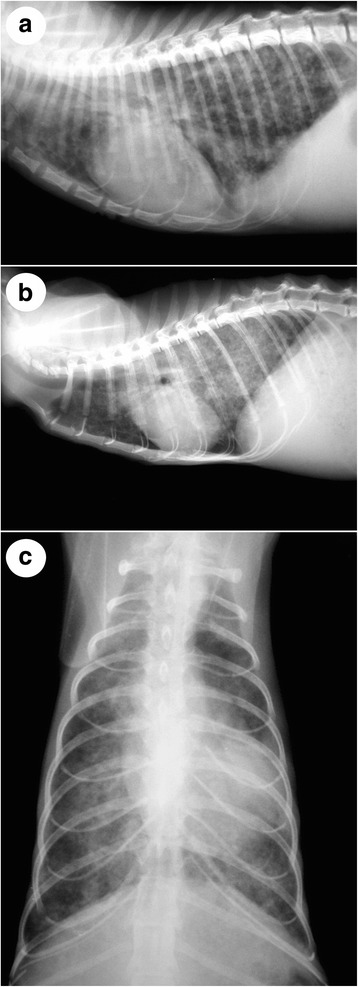
Thoracic radiographs of cats infected with *Aelurostrongylus abstrusus*: **a** lateral thoracic radiograph from a 3-year-old female cat with moderate dysponea and coughing. There is a generalised alveolar-interstitial pattern; **b** and **c** lateral and dorso-ventral radiographs from a 1-year-old male cat, living outdoors with severe aelurostrongylosis. The cat presented with cachexia, coughing, severe dysponea, and died 3 days after examination. There is a significant interstitial-alveolar pattern, affecting the diaphragmatic lung lobes in particular

Radiographic findings are not necessarily pathognomonic for aelurostrongylosis, but evidence of pulmonary interstitial disease is often evident (Fig. 3). Clinical suspicion can result from thoracic radiography and findings depend on the stage of infection, infection dose, and the stage of the disease (acute or chronic) [65]. In experimental settings, bronchial and focal alveolar patterns are usually observed in the first stage of disease and bronchointerstitial patterns are visible after partial resolution of alveolar disease [69]. Interestingly, no signs of pulmonary hypertension or an associated right ventricular response were detected [89]. Naturally infected symptomatic cats might also show a mix of bronchial and interstitial pulmonary patterns in thoracic radiographs [57]. In two infected cats, multiple areas of opacity and increased bronchial diameter, more accentuated in the caudal lung lobe have been revealed by computed tomography (CT) [80]. In another experimental study, multifocal nodules of various sizes were observed throughout the lungs, affecting all lung lobes, and, with disease progression, peripheral areas with an alveolar pattern increased probably due to accumulation of eggs, larvae and inflammatory debris in the alveoli [65]. CT was able to assess bronchial thickness quantitatively and identified enlarged lymph nodes. Also, characteristic CT findings illustrated that changes due to *A. abstrusus* infections were consistent with histopathological findings [65]. By enabling the detection of small lesions and the differentiation of superimposed structures, CT represents a highly valid tool to evaluate the extent of damages due to *A. abstrusus* infections and the corresponding prognostic features.

### Gross pathology and histopathological findings

#### Gross morphology

**Fig. 4:**
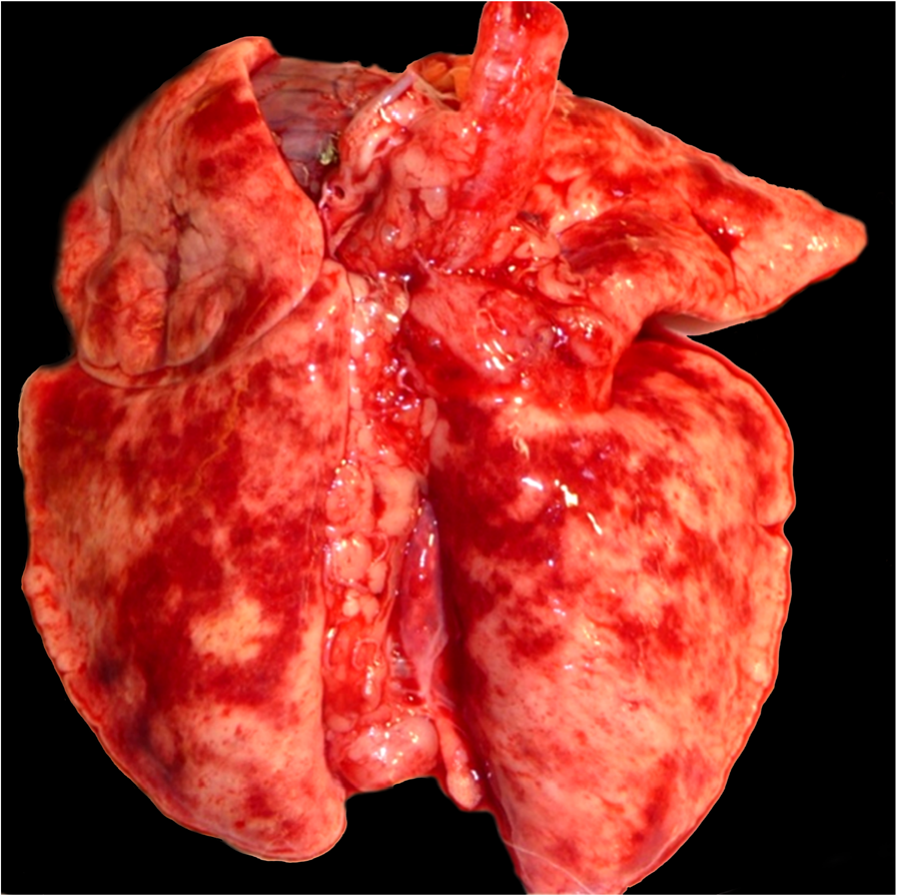
Lung of a male (neutered), 1-year-old European shorthair cat infected with *Aelurostrongylus abstrusus*. White-greyish irregularly shaped areas of consolidation are randomly distributed over the whole lung and are interspersed with dark red, hyperaemic areas. The affected areas are multifocal, locally extensive to coalescent and when sliced of caseous nature. Also, lung lymph nodes are enlarged

As shown in Fig. 4 lungs of naturally infected cats showed lesions that varied from multiple small foci of pale pink colour [61] to large consolidated and congested areas, whose colour varied from mottled dark to light brown [58] and from focal grey to white [15]. Occasionally, the discharge of foamy [61] to caseous [63] liquid was observed. Comparable findings were detected in experimentally infected cats, where irregularly distributed consolidated nodules brownish to greyish colour with adjacent dark red hyperaemic areas, were observed in the lung 12 weeks after infection. These nodules were observed to protrude from the lung surface. In less affected cats single small dark-red areas were present. Also, pale and irregular corridors were observed on the surface [14]. The presence of enlarged lung and tracheobronchial lymph nodes was a constant feature [14, 62]. A description of the sequential pathological changes that occur in the lung during and after prepatency has previously been described in details [64].

#### Histopathology

**Fig. 5:**
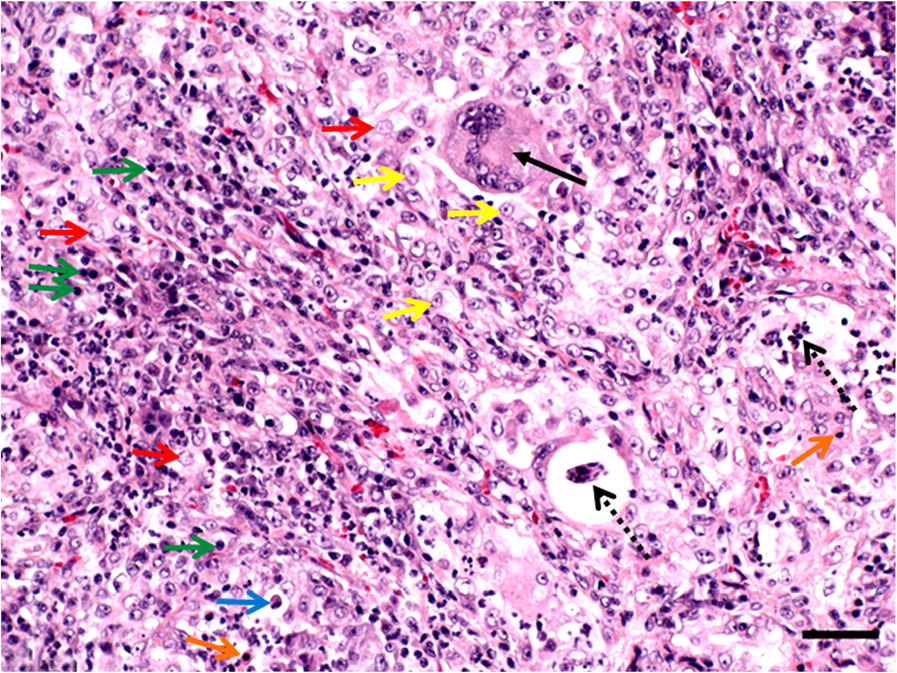
Histopathological examination of a cat lung infected with *Aelurostrongylus abstrusus*. Macroscopic consolidated areas correspond histologically to lung tissues presenting massive cellular infiltration: lymphocytes (*green arrows*), macrophages (*red arrows*), multinucleated giant cells (*black arrow*), epithelioid histiocytes (*yellow arrows*) as well as eosinophils (*orange arrows*) and plasma cells (*blue arrow*) are densely packed forming granulomas. Alveolar lumina are obliterated and sections of parasitic eggs and larvae (*dotted black arrows*) are visible. Haematoxylin and eosin. *Scale-bar*: 50 μm

The most classical picture observed in cats with aelurostrongylosis is the presence of numerous eggs and larvae at various stages of development in the alveoli and bronchi [58, 61, 63]. Adult stages are rarely present in small bronchioles [58, 61]. Worm eggs infiltrating the interstitial tissue and bronchioles are surrounded with inflammatory cells (Fig. 5), mainly lymphocytes, eosinophils, macrophages and giant cells [58, 61, 63]. Inflammatory response leads to thickening of the interstitial tissue, along with oedema and haemorrhage [63]. The bronchial epithelium also becomes hyperplastic and hyperactive bronchiolar glands contribute to increased mucus production [63] and the peribronchial lymph nodes show follicular hyperplasia [63, 67]. A marked hypertrophy and hyperplasia of the muscles of the terminal bronchioles [63, 67], associated with muscular hypertrophy of the walls of the alveolar ducts [67] and hypertrophy and hyperplasia of the media muscle of the pulmonary arteries [68] and several alterations in the ultrastructure of pulmonary arteries have been described in cats [62, 90]. Detailed descriptions of the progressive pathological alterations that occur in experimentally infected cats have been previously reported [14, 62, 64].

### Treatment of feline aelurostrongylosis

#### Mild to moderate cases

In general, the use of anthelmintic treatment is sufficient to resolve the clinical signs. Topical parasiticides are an easy-to-apply choice for treating *A. abstrusus* infections, because of safety and ease of administration, especially when multiple dosing is required and feral or indocile cats are difficult to handle and manage. A number of anthelmintic spot-on preparations licensed for the use in cats have demonstrated a high potential for *A. abstrusus* efficacy. In particular, the emodepside 2.1 %/praziquantel 8.6 % spot-on solution is licensed in some markets (e.g. Australia) to treat cat aelurostrongylosis and proved to be 99.38 % and 100 % effective in stopping larval shedding and in curing clinical signs, respectively [91, 92]. Additionally, the adulticidal efficacy of emodepside has been investigated in two randomized, placebo-controlled experiments, which have demonstrated that two spot-on administrations of the molecule 2 weeks apart are safe and at least 99.2 % effective in eliminating adult worms in treated cats [93]. Fenbendazole is licensed in some countries (e.g. UK) for treating *A. abstrusus* infection and has been shown to be efficacious when administered orally at 50 mg/kg body weight (BW) for three consecutive days with an efficacy greater than 99 % for larval count reductions [56, 91, 92, 94]. Fenbendazole was also successfully used to eliminate *A. abstrusus* in a domestic shorthair cat when administered at 50 mg/kg PO q24h for 14 days as evidenced by improvement in blood parameters, enhanced clinical recovery and absence of *A. abstrusus* larvae by Baermann fecal examination 2 weeks after treatment [76].

The spot-on combination containing imidacloprid 10 %/moxidectin 1 % is currently licensed in some markets (e.g. Australia) and moxidectin has been shown to have high efficacy in naturally infected cats, approaching 100 % for reduction of larval excretion [91, 92]. However, the efficacy was assessed based on the cessation of larval shedding, which can be limited by the sensitivity of the detection method and the intermittent shedding of L1s in infected cats. The novel formulation containing eprinomectin 0.4 % w/v in combination with fipronil 8.3 % w/v, (S)-methoprene 10 % w/v and praziquantel 8.3 % w/v in a spot-on solution has also been evaluated against *A. abstrusus* in natural and experimental infections. In particular, using time points of treatment selected based on the endogenous cycle of *A. abstrusus* in the cat, i.e. from infective L3s to adult stages; it has been experimentally shown that single treatments with eprinomectin had a high efficacy against all *A. abstrusus* stages concerning stopping of larval shedding, e.g. 99.6 % efficacy in cats treated 32 days after inoculation (and therefore harbouring adult parasites) [95]. In naturally infected cats the same formulation was demonstrated to be safe and efficacious in achieving a faecal larval reduction of 90.5 % [96].

Furthermore, oral milbemycin oxime 4 mg/kg (plus praziquantel 10 mg/kg BW) at 2-week intervals was also effective in stopping larval shedding and resulted in resolution of clinical signs over a period of 6 weeks in a single cat with a clinical aelurostrongylosis [72]. Selamectin used topically at 18 mg/kg BW was able to reduce clinical signs after a single spot-on administration, while a second dose after 1 month ensured improvement in respiratory function and radiographic bronchial lesions [97]. The same molecule at a topical dose of 6 mg/kg BW was effective in eliminating L1s from the faeces of a cat after 30 days [57], and when applied to ten adult cats four times in 2 months clinical signs improved and larval shedding stopped in nine cases [98].

Levamisole has also been demonstrated to be an efficacious and safe treatment for *A. abstrusus* [99], but there are no commercial licensed preparations for cats containing this drug. Toxicity concerns exist with the use of *off label* injectable ivermectin in cats, especially kittens [100]. About 2 to 3 weeks after the completion of anthelmintic treatment, efficacy of treatment should be confirmed through Baermann technique performed on faecal samples for three consecutive days.

#### Severe cases

Adequate control of inflammation and prompt detection of associated complications are crucial in order to improve the overall prognosis of the disease. Hence, in severe clinical cases supportive treatment is needed, for instance in cases complicated with secondary bacterial infection and inflammatory reactions, broad-spectrum antibiotics should be administered together with anti-inflammatory doses of corticosteroids (e.g. prednisolone 0.5 mg/kg PO q24h for 10 days) [76, 101]. Antibiotics should be selected on the basis of culture and sensitivity, with doxycycline being a good choice where concurrent *Bordetella bronchiseptica* or *Mycoplasma* spp. infections are involved. If respiratory tract congestion is present, then a mucolytic, such as bromhexine may help to ease associated discomfort and dyspnoea. Bronchodilators, such as theophylline or terbutaline may also be useful in treating severe dyspnoea. Heavily affected cats with respiratory distress could benefit from supportive oxygen administration, and when pleural effusion and pneumothorax are observed, immediate thoracocentesis is recommended [101].

### Prevention of feline aelurostrongylosis

Eradication of *A. abstrusus* is impractical in any given area, as significant reservoirs are present in intermediate hosts as well as feral and stray cat populations. While theoretically molluscicides could be employed to reduce slug and snail numbers, their use should be discouraged because they may be toxic for pets and the environment. Although there are pet-safe molluscicides, such molluscicide treatments may be ineffective due to continuous fresh snail and slug migration into the concerned areas and to the free-ranging nature of cats. Avoiding predation by keeping cats indoors is therefore currently considered the only potential way to avoid infection [101]; however, this is not recommended for animal welfare reasons. Spot-on preparations containing emodepside, eprinomectin, moxidectin and selamectin have all been demonstrated to eliminate larval shedding by at least 90 % [91, 92, 95, 96, 98]. Therefore, their use as part of a wider parasite control strategy or in cats that are at high risk of infection is likely to reduce parasite transmission and possibly have some use as a prophylactic measure. An efficacious treatment of infected cats may significantly reduce the environmental contamination with faecal larvae and, as a consequence, the number of infected intermediate and paratenic hosts. However, this is only theoretical because, besides concerns regarding reasonability and necessity of preventive treatments and financial requirements, there is likely to be a consistent reservoir in stray or feral cats, which are also the animals with the highest rates of infection, contributing to the maintenance of the life-cycle of *A. abstrusus*.

The efficacy of eprinomectin administered against pre-patent developmental stages shown in experimental studies is promising in the chemoprevention of aelurostrongylosis because it proved to limit the progressive pulmonary changes that occur during the infection [95]. Moxidectin is another potential option for the chemoprevention of aelurostrongylosis. This molecule remains at detectable levels for weeks after treatments [102] and consistent administrations of topical moxidectin can induce elevated and sustained steady-state plasma concentrations [103]. Studies evaluating the efficacy of moxidectin steady-state in protecting from subsequent infection by *A. abstrusus* (and other lungworms as well) would be useful for further approaches in the prevention of these infections.

### Research challenges and needs

Feline aelurostrongylosis is an underappreciated, mostly neglected illness. This poses a challenge and highlights significant research areas that are indispensable to addressing aelurostrongylosis in the coming decades; these areas are discussed below.

#### Diagnostics

Feline aelurostrongylosis is a disease that is pervasive in both the developing and developed regions. While feline aelurostrongylosis may be self-limiting [1, 57], identification of the aetiological agent is required for the management of diseased cats. Major problems in the diagnosis of aelurostrongylosis include its non-specific clinical presentation and the lack of sensitive diagnostic methods. Baermann-based diagnosis is actually the gold standard, although this method has its own limitations, such as occasional difficulties in differentiating L1s from altered larvae in faecal samples, resulting in false-negative results and, importantly, the difficulty in obtaining fresh faecal samples from cats having outdoor access. PCR-based detection of *A. abstrusus* are relevant for research purposes, but recent data reveal that PCR-based ITS sequencing was promising for identification of *A. abstrusus* from various types of clinical specimens [52]. Serological diagnostics are still in the starting blocks, but promising results indicate that detection of antibodies using ELISA might be a useful tool for mass-screening and seroepidemiological studies, and also potentially for individual diagnosis [88]. Nonetheless, improving existing assays and developing new technologies that offer increased sensitivity, specificity, availability and/or efficiency is warranted. Currently, in the absence of an optimal diagnostic technique, simultaneous application of PCR (from faeces or material obtained from tracheal swabs or BAL) and the Baermann’s method could be employed for effective detection of *A. abstrusus* infections.

#### Molecular epidemiology

In recent years the clinical significance and wide recognition of metastrongyloid nematodes such as *A. abstrusus* is becoming apparent. It would be useful to investigate prevalence of infection in domestic and wild felid populations in countries where recent data are lacking. Interestingly, a recent study has detected *A. abstrusus* in 6 out of 21 wild large felids housed in sanctuaries and protected areas of South Africa [104]. The study provided the first definitive evidence of the ability of *A. abstrusus* to infect lions (*Panthera leo*) and was the first to report aelurostrongylosis in servals (*Leptailurus serval*) and caracals (*Caracal caracal*). Epidemiology of *A. abstrusus* in the feline definitive host and intermediate gastropod hosts has been extensively studied. However, knowledge of animals that serve as intermediate and paratenic hosts of *A. abstrusus* in different geographical regions is still poor. Also, host-pathogen relationships and the population structure of *A. abstrusus* in feral and domestic cats have not been well defined on a wide range of spatial and/or temporal scales. Population genetics and phylogenetics have the potential to provide new insights into the epidemiology and ecology of feline aelurostrongylosis. The complete mitochondrial genome sequence was a significant start in this regard [54]. Nevertheless, many questions remain to be addressed. For example, the degree of genetic similarity between *A. abstrusus* strains from wild and domestic felids is unknown. Sympatric feral and domestic felids can potentially shed larvae of different genotypes, and the contributions of these hosts to environmental parasite load have not been defined.

The distribution of different *A. abstrusus* genotypes in different ecosystems could have implications for parasite transmission cycles and the potential for different gastropods and paratenic hosts to contribute to infection of felids. Further, analysis of *A. abstrusus* strains in a large sample of geographically and temporally overlapping domestic and wild felids would provide important insights into any co-existing domestic and sylvatic cycles of *A. abstrusus*. Molecular characterization is the basis to infer population structure, gene flow (i.e. between host populations and between different geographical locations) and to predict the evolutionary dynamics of *A. abstrusus*.

#### Immunopathology and pathogenesis

Unfortunately, the number of studies that examined the immune response in aelurostrongylosis is limited and results have been contradictory. An earlier study suggested that immunity might be an important element of pathogenesis as indicated by the cessation of the production of larvae and protection conferred after repeated infections [66]. However, results obtained in another study showed that 56 % of re-infected cats resumed the shedding of larvae in the faeces, although exhibiting a longer pre-patent period compared to cats with a single infection [20]. Also, the correlation between acquired immunity and the level of infection in naturally infected cats could not be established [15]. Further, little is known about the potential heterogeneity in the immunological responses of individual cats infected with *A. abstrusus*, which may account for the broad spectrum of clinical manifestations. An interesting area that requires investigation in this respect is the contribution of host genetic and parasite genetic factors to the severity of aelurostrongylosis in infected cats, because possible *A. abstrusus* genotypes may play a different role in virulence and may impact the animal immunological response to infection.

Like many other helminth parasites, *A. abstrusus* can cause chronic infection and infected cats may harbour worms in their lungs for years without excreting L1s in faeces. This long-term form of infection and the survival of *A. abstrusus* within the feline host indicate that this parasite must have developed some mechanisms to evade the cytotoxic effects of the host immune response. In helminth infections, immune response is often dominated by the production of T helper type 2 (Th2) immune cytokines, such as interleukin-4 (IL-4), IL-5 and IL-13, which represent a critical immune response against helminths invading cutaneous or mucosal sites, such as *A. abstrusus*; these also may play a role in reducing the severity of acute illness. Th2 inflammatory responses are characterized by the recruitment and activation of mast cells, basophils and eosinophils, and goblet cell hyperplasia in airway and intestinal epithelia [105]. Bronchoalveolar lavage analysis of *A. abstrusus*-experimentally infected cats revealed a significant increase in the number of eosinophils, macrophages and neutrophils following infection [106]. Eosinophilia has been found to be important in controlling migrating larvae of the nematode *Nippostrongylus brasiliensis* at the lung and intestinal stages [107]*.* Thus, high levels of eosinophils associated with *A. abstrusus* infection may play an important role in the inflammatory response of Th2 cells during feline aelurostrongylosis, although not necessarily increased in peripheral blood [14]. A better knowledge of the humoral immune response against *A. abstrusus* at different stages of infection is required to permit significant advances in this domain. Other important aspects of pathogenesis that are not well understood include the onset of acute phase proteins, being part of the innate immune response and the protein profiles in *A. abstrusus* infected cats. Identifying and quantifying alterations in serum protein of infected cats may elucidate the immuno-inflammatory pathways that operate during infection and can be used as potential diagnostic and prognostic biomarkers.

A greater understanding of these issues should allow us to bridge the gap in understanding of the epidemiology and genetic diversity of *A. abstrusus*, and to resolve contradictory observations in immune-pathogenesis of *A. abstrusus* infections. This will subsequently allow us to track the transmission pathways and the dynamics of *A. abstrusus*, and to better understand factors influencing disease pathogenesis. Ultimately, all facets of *A. abstrusus* research will enable earlier disease diagnosis, better surveillance, and will lead to the development of more tailored control and treatment measures.

## Conclusions

Cat aelurostrongylosis is one of the most important parasitic diseases that is likely to continue to threaten feline health and welfare in the years to come. Although the possible wide host range of this parasite in domestic and wild felid species has been described, further studies are necessary to elucidate the relative contribution of different feline species to *A. abstrusus* transmission in different countries and the potential for transmission maintenance in each species in the absence of other definitive host populations. There is no doubt that there will continue to be a shifting landscape in the host-specificity of *A. abstrusus* in the decades to come. Also, sequencing of the mitochondrial genome has been a milestone in unraveling the phylogenetic position of *A. abstrusus*. However, the availability of more genome sequences will advance our knowledge of the molecular epidemiology and the genetics of *A. abstrusus*, and will have great importance for the development of molecular assays that can provide accurate taxonomic knowledge, including clear species boundaries and more resolved phylogenies. Humoral immune response seems to be more important than cell-mediated immunity for feline host defense against disease caused by *A. abstrusus*. However, immunopathogenesis as a core mechanism to the broad spectrum of clinical signs observed in infected cats remains understudied. Likewise, the underlying mechanisms of heterogeneity in the immunological responses of individual cats infected with *A. abstrusus*, particularly potential genetic mechanisms, have not been investigated. The next decade promises new opportunities to understand the genetic susceptibility and immunological variations to *A. abstrusus* infection. This knowledge combined with a deepened understanding of innate, humoral, and cell-mediated immunity to *A. abstrusus* infection has potential for guiding new opportunities for development of new diagnostics and more efficient treatment and prevention of aelurostrongylosis.

## Abbreviations

BAL, bronchoalveolar lavage; COX1, cytochrome *c* oxidase subunit I; CT, computed tomography; ELISA, enzyme-linked immunosorbent assay; ETC, external transcribed spacer; IFAT, indirect fluorescent antibody test; IL-4, interleukin-4; ITS1, internal transcribed spacer 1; L1s, first-stage larvae; L3s, third-stage larvae; rDNA, ribosomal DNA; Th2, T-helper type 2
